# The complex role of AIM2 in autoimmune diseases and cancers

**DOI:** 10.1002/iid3.443

**Published:** 2021-05-20

**Authors:** Huan Zhu, Ming Zhao, Christopher Chang, Vera Chan, Qianjin Lu, Haijing Wu

**Affiliations:** ^1^ Department of Dermatology, Hunan Key Laboratory of Medical Epigenomics The Second Xiangya Hospital of Central South University Changsha China; ^2^ Division of Rheumatology, Allergy and Clinical Immunology University of California at Davis School of Medicine Davis California USA; ^3^ Division of Rheumatology and Clinical Immunology, Department of Medicine The University of Hong Kong Hong Kong China; ^4^ Institute of Dermatology Chinese Academy of Medical Sciences and Peking Union Medical College Nanjing China

**Keywords:** AIM2, autoimmune diseases, cancers, cGAS‐STING

## Abstract

Absent in melanoma 2 (AIM2) is a novel member of interferon (IFN)‐inducible PYHIN proteins. In innate immune cells, AIM2 servers as a cytoplasmic double‐stranded DNA sensor, playing a crucial role in the initiation of the innate immune response as a component of the inflammasome. AIM2 expression is increased in patients with systemic lupus erythematosus (SLE), psoriasis, and primary Sjogren's syndrome, indicating that AIM2 might be involved in the pathogenesis of autoimmune diseases. Meanwhile, AIM2 also plays an antitumorigenesis role in an inflammasome independent‐manner. In melanoma, AIM2 is initially identified as a tumor suppressor factor. However, AIM2 is also found to contribute to lung tumorigenesis via the inflammasome‐dependent release of interleukin 1β and regulation of mitochondrial dynamics. Additionally, AIM2 reciprocally dampening the cGAS‐STING pathway causes immunosuppression of macrophages and evasion of antitumor immunity during antibody treatment. To summarize the complicated effect and role of AIM2 in autoimmune diseases and cancers, herein, we provide an overview of the emerging research progress on the function and regulatory pathway of AIM2 in innate and adaptive immune cells, as well as tumor cells, and discuss its pathogenic role in autoimmune diseases, such as SLE, psoriasis, primary Sjogren's syndrome, and cancers, such as melanomas, non‐small‐cell lung cancer, colon cancer, hepatocellular carcinoma, renal carcinoma, and so on, hopefully providing potential therapeutic and diagnostic strategies for clinical use.

## Graphical abstract

Herein, we provide an overview of the emerging research progress on the function and regulatory pathway of absent in melanoma 2 (AIM2) in innate and adaptive immune cells, as well as tumor cells, and discuss its pathogenic role in autoimmune diseases, such as systemic lupus erythematosus, psoriasis, primary Sjogren's syndrome, and cancers, such as melanomas, non‐small‐cell lung cancer, colon cancer, hepatocellular carcinoma, renal carcinoma, and so on, hopefully providing potential therapeutic and diagnostic strategies for clinical use.

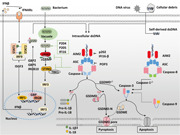

## 1 INTRODUCTION

Absent in melanoma 2 (AIM2), discovered in melanoma in 1997,^1^ was originally described as a novel member of interferon (IFN)‐inducible PYHIN proteins, which contains four members in humans (AIM2, IFI16, IFIX, and MNDA) and 13 homologous proteins (e.g., Aim2, p202, p204, and p205) in mice.^2^ In 2009, four groups independently found that AIM2 is capable of recognizing cytosolic double stranded DNA (dsDNA) of pathogens‐associated or host origin, recruiting apoptosis‐associated speck‐like protein containing a CARD (ASC) and pro‐caspase‐1, and inducing caspase‐dependent inflammasome formation, thereby triggering mature interleukin 18 (IL‐18) and IL‐1β production or leading to gasdermin‐D (GSDMD)‐mediated pyroptosis,^3^, ^4^, ^5^, ^6^ which eventually results in the initiation of an innate immune response against pathogens invasion.^5^ However, abnormal activation of AIM2‐mediated immune response in response to cellular perturbations has been reported to cause immune‐linked disorders such as systemic lupus erythematosus (SLE),^7^ psoriasis,^8^ primary Sjogren's syndrome,^9^, ^10^ and polyarthritis.^11^ However, the underlying mechanism is poorly understood.

AIM2 was originally described as a tumor suppressor for melanoma.^1^ Recent developments have indicated that AIM2 contributes to the tumorigenesis of non‐small‐cell lung cancer (NSCLC) via regulation of mitochondrial dynamics and inflammasome activation.^12^ An inflammasome‐independent pathway of AIM2 has also been discovered, controlling intestinal cell proliferation, apoptosis, and metastasis in the regulation of colon cancer through suppression of AKT activation.^13^ Therefore, dysregulation of AIM2 plays a multifarious role in the pathogenesis of tumors. Indeed, tumor‐suppressive properties of AIM2 have been identified in hepatocellular carcinoma,^14^ renal carcinoma,^15^ breast cancer,^16^ colon cancer,^13^ HPV‐infected cervical carcinoma,^17^ and prostate cancer.^18^ The absence of AIM2 promotes hepatocellular carcinoma metastasis, while overexpression of AIM2 induces breast cancer cell apoptosis.^14^, ^16^ More recently, activation of AIM2 was also shown to exert a carcinogenic effect in NSCLC,^12^ squamous cell carcinoma (SCC),^19^, ^20^ and Epstein‐Barr virus‐associated nasopharyngeal carcinoma (EBV‐associated NPC).^21^

In this review, we systemically summarize the current research progress on the function of AIM2 and discuss its pathogenic role in autoimmune diseases and tumors, providing a better understanding of disease pathogenesis and potential diagnostic and therapeutic targets.

## 2 ASSEMBLY AND ACTIVATION OF THE AIM2 INFLAMMASOME

**Figure 1:**
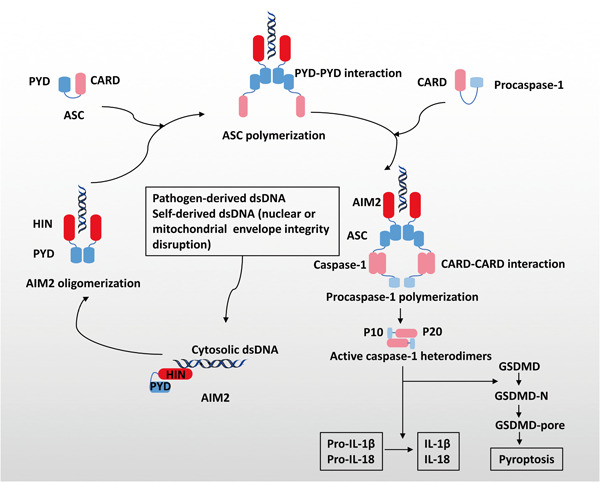
Assembly and activation of the AIM2 inflammasome. Sensing abnormal dsDNA in the cytoplasm triggers the assembly of the AIM2 inflammasome. The adapter protein ASC and the effector protein procaspase‐1 are recruited to participate in this activation process. AIM2, absent in melanoma 2; ASC, apoptosis‐associated speck‐like protein containing a CARD; dsDNA, double stranded DNA; GSDMD, gasdermin‐D; IL, interleukin

The AIM2 proteins consist of two main domains: a C‐terminal HIN domain and an N‐terminal pyrin domain (PYD) (Figure 1).^22^ Upon sensing pathogen‐derived dsDNA or release of dsDNA secondary to disruption of the integrity of the nuclear or mitochondrial envelope, the HIN domain directly recognizes dsDNA through its two oligonucleotides/oligosaccharide‐binding folds in a sequence‐independent manner. The PYD domain forms the structural template via ligand binding and oligomerization and interacts with the PYD of a recruiting adapter protein ASC, resulting in ASC polymerization.^4^, ^5^, ^6^, ^23^, ^24^, ^25^ Inactive zymogen procaspases‐1 are subsequently recruited into these multimolecular complexes via the CARD–CARD interaction, and cleaved into heterodimers consisting of a small subunit p10 and a large subunit p20.^26^ Active caspase‐1 eventually leads to proteolytic cleavage of IL‐1β, IL‐18, and caspase substrate GSDMD, whose N‐terminal polymerizes at the cell membrane forming the GSDMD pores and mediating pyroptosis.^26^, ^27^, ^28^, ^29^, ^30^ Activation of the AIM2 inflammasome is a double‐edged sword. It provides immunosurveillance for foreign pathogens by the initiation of the innate immune response, and it maintains central nervous system homeostasis during normal nerve development by GSDMD‐mediated pyroptosis.^31^ However, abnormal activation can also lead to tissue injury and inflammation such as that induced by ionizing radiation.^32^

## 3 ROLE OF AIM2 IN IMMUNE CELLS

Since AIM2 is reported as a component of the inflammasome, the function and regulatory pathway of AIM2 in immune cells, especially in innate immune cells, has been intensively studied (Table 1). In the following paragraphs, we will summarize the role of AIM2 in innate and adaptive immune cells in detail.

**Table 1:** Role of AIM2 in immune cells

Cell types	Role of AIM2	References
Macrophages	AIM2 inflammasome activation leads to antimicrobial host immune responses	3, 4, 5, 27, 28, 33
	AIM2 inflammasome over‐activation leads to abnormal functional maturation of macrophages thereby facilitating the progression of SLE	
	AIM2 inflammasome activation leads to high levels of IL‐18 secretion from Kupffer cells thereby promoting hepatic NK cell activity and increasing NK cell‐dependent IFN‐γ	
	Induction caspase 1‐mediated cleavage of cGAS thereby upregulating PD‐L1 and IDO, which inhibit antibody‐dependent cellular cytotoxicity and T cell‐mediated cytotoxicity to evade the adaptive response	
DCs	AIM2 inflammasome activation leads to antimicrobial host immune responses	34, 35, 36, 37
	AIM2 inflammasome participates in the process of CD137L‐mediated monocyte to DC differentiation	
	AIM2 inflammasome activation leads to high levels of IL‐1α production thereby promoting lung cancer cell proliferation	
Neutrophils	Expression of key components of AIM2 inflammasome	^38^
T cells	Induction antigen‐specific antibody response thereby enhancing adaptive immunity of CD8^+^ T cells	39, 40, 41, 42, 43
	Upregulation of AIM2 in Treg restrain autoimmune diseases by reducing AKT–mTOR signaling and a T cell‐intrinsic role	
B cells	Upregulation of AIM2 in gastric B cells inhibits CXCL16 production in the control of infiltration and retention of CD8^+^ T cells within chronic inflammatory tissues	44, 45
	Upregulation of AIM2 preferential in mature memory CD27^+^ B cells of adults	
	Downregulation of AIM2 by FOXP1 in mature human B cells	

Abbreviations: AIM2, absent in melanoma 2; DCs, dendritic cells; IFN‐γ, interferon γ; IL, interleukin; SLE, systemic lupus erythematosus.

### 3.1 AIM2 in macrophages

**Figure 2:**
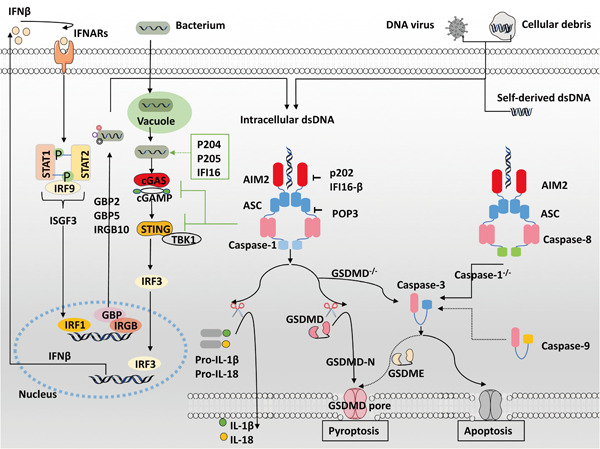
Regulation of the AIM2 inflammasome in macrophages. Cytosolic dsDNA from pathogens or host cells is sensed by AIM2, which attracts ASC and procaspase‐1 to trigger mature IL‐18 and IL‐1β production or GSDMD‐mediated pyroptosis. In GSDMD‐deficient macrophages, AIM2 induces caspase‐1‐dependent caspase‐3 activation whereas AIM2 drives caspase‐8‐dependent caspase‐3 activation without caspase‐1, resulting in apoptosis. The AIM2 inflammasome negatively regulates the cGAS/STING‐driven type I IFN response and can be inhibited by the IFN‐inducible proteins POP3 and IFI16‐β in humans or p202 in mice. AIM2, absent in melanoma 2; ASC, apoptosis‐associated speck‐like protein containing a CARD; dsDNA, double stranded DNA; GSDMD, gasdermin‐D; IFN, interferon; IL, interleukin

Macrophages play an essential role in the innate immune response by engulfing pathogens and cellular debris, presenting antigens, and producing cytokines.^46^ Numerous references in the literature confirm that phagocytosed pathogens‐ or self‐derived dsDNA can be recognized by AIM2 in macrophage cytosol, triggering caspase‐1‐dependent IL‐18 and IL‐1β release or leading to GSDMD‐mediated pyroptosis (Figure 2).^3^, ^4^, ^5^, ^27^, ^28^, ^33^ Additionally, the AIM2 inflammasome also drives apoptosis through caspase‐1‐mediated caspase‐3 activation in GSDMD^−/−^ macrophages,^47^, ^48^ or caspase‐8‐mediated caspase‐3 activation in caspase‐1^−/−^ macrophages.^49^, ^50^, ^51^ Recent studies show that caspase‐9 is also involved in this apoptosis pathway to induce caspase‐3 activation, which in turn cleaves GSDME inducing secondary necrosis or pyroptosis.^47^, ^52^, ^53^

Copious evidence indicates the activation of AIM2 inflammasome involved in the elimination of macrophages infected with intracellular pathogens such as *Francisella tularensis*,^34^, ^35^, ^36^ *Mycobacterial* species,^54^, ^55^ *Listeria monocytogenes*,^56^, ^57^, ^58^ *Brucella abortus*,^59^ *Legionella pneumophila*,^60^ *Streptococcus pneumonia*,^61^ *vaccinia virus*,^4^ *murine cytomegalovirus*,^36^ and *Toxoplasma*.^50^ Aim2^−/−^ mice are prone to infections. For example, Aim2^−/−^ mice challenged with *cytomegalovirus* exhibit greater viral titers than wild‐type mice.^36^ Aim2‐deficient mice are exceedingly susceptible to infections with *Francisella tularensis* or *Mycobacterium tuberculosis* and have a higher bacterial burden than wild‐type mice.^34^, ^35^ Bone marrow‐derived macrophages from these mice fail to generate an inflammasome.^34^, ^35^, ^54^, ^62^ This indicated in some cases AIM2 is indispensable for a complete antimicrobial host response.^35^, ^36^ Besides, macrophages lacking type I IFN response impair AIM2 inflammasome activation.^34^, ^63^, ^64^ This implies efficient activation of the AIM2 inflammasome requires the presence of type I IFN signaling during bacterial infections.^35^, ^59^, ^61^, ^63^, ^64^, ^65^

It has been recognized that bacterial DNA sensed by other cytosolic sensors, such as cGAS, leads to the binding and trafficking of STING and TANK‐binding kinase 1 (TBK1),^66^ thus inducing transcription factor IRF3 phosphorylation and IFN‐β production.^63^, ^67^, ^68^, ^69^ IFNβ then combines with type I IFN receptors (IFNARs),^65^ forming IFN‐stimulated gene factor 3 (ISGF3) complexes, inducing transcription factor IRF1 expression and further upregulating immunity‐related GTPase (IRG) and guanylate‐binding protein (GBP).^63^, ^64^, ^70^, ^71^ The combination of GBP2 and GBP5 with IRGB10 promotes bacteriolysis and subsequent complete activation of AIM2 inflammasome.^63^, ^64^, ^71^, ^72^, ^73^ However, type I IFN signaling also upregulates TNF‐related apoptosis‐inducing ligand (TRAIL) to activate apoptotic caspases and cause cell death, which is detrimental in vivo during *Francisella* infections.^67^, ^70^ Previous studies have shown that the AIM2 inflammasome inhibits the type I IFN pathway.^74^ It is likely that the AIM2 inflammasome negatively regulates cGAS/STING‐driven type I IFN activity by depleting intracellular potassium, hampering STING and TBK1 binding, and inducing caspase 1‐dependent cleavage of cGAS.^62^, ^74^, ^75^, ^76^, ^77^

Macrophage phagocytosis contributes to tumor elimination.^78^ However, the antitumor effects during antibody treatment also lead to a concomitant undesired effect. Macrophages can lead to immunosuppression of HER2^+^ breast cancers after antibody‐dependent cellular phagocytosis, in which AIM2 inflammasome plays a vital role.^77^ Mechanistically, AIM2 dampens the cGAS–STING pathway by upregulating compensatory immunosuppressive checkpoints PD‐L1 and IDO, which inhibit antibody‐dependent cellular cytotoxicity and T cell‐mediated cytotoxicity to evade the adaptive response.^77^ Thus, AIM2 inflammasome in macrophages can initiate the innate immune response, and participate in escape adaptive immune response for tumor cells.

#### 3.1.1 The regulation of AIM2 in macrophages

AIM2 inflammasome pathway is tightly regulated in cells and affected by posttranslational modifications. The assembled inflammasomes in macrophages trigger the G protein RalB and undergo degradation by ubiquitinated TRIM11 via p62‐dependent selective autophagy.^79^, ^80^ Also, the mitochondrial serine protease HtrA2 regulates the duration and magnitude of AIM2 inflammasome activity in a protease activity‐dependent manner.^81^

The upregulation of 25‐hydroxycholesterol in activated macrophages is required to restrain abnormal AIM2 inflammasome activation.^82^ Human POP3 directly binds to AIM2 and dampens inflammasome activation.^83^ Additionally, several other proteins within the IFN‐inducible PYHIN family have a close connection to AIM2 inflammasome activation signaling. IFI16‐β, a DNA sensor, inhibits AIM2‐mediated dsDNA sensing in human THP‐1 cells.^84^ Similar to IFI16‐β, mouse p202, a lupus susceptibility factor, interacts with AIM2 and halts dsDNA‐dependent caspase activation.^3^ Mouse p205 activates STING‐driven type I IFN signaling, whereas AIM2 dampens this pathway, likely by sequestering p205 from STING.^85^ Mouse p204 or its human ortholog IFI16 are also involved in type I IFN production,^67^, ^86^ whereas the AIM2 inflammasome inhibits type I IFN signaling. Therefore, the relationship between AIM2 and the regulatory protein response to cytoplasmic DNA needs further understanding in the context of various diseases.

### 3.2 AIM2 in dendritic cells

Dendritic cells “swallow” pathogens by phagocytosis and migrate to lymphoid tissue, where they present processed antigens to T cells.^87^, ^88^ Similar to macrophages, activation of AIM2 inflammasome in dendritic cells (DCs) triggers an innate immune response against pathogen infection, including infection with *Francisella novicida*,^36^, ^37^ *Mycobacterial* species,^62^ and *Adenovirus*.^89^ However, interactions of the AIM2 inflammasome and type I IFN pathway within macrophages do not always mirror their crosstalk within DCs. For example, IFN‐β signaling is indispensable for inflammasome activation within macrophages, while it is partially required in DCs infected with *Francisella novicida*.^34^, ^35^, ^36^, ^37^ Additionally, AIM2 inflammasome activation and IL‐1β secretion are involved in the process of CD137L‐mediated monocyte to DC differentiation, which triggers a stronger T cell response against cancer‐associated viruses.^90^ In contrast, AIM2 inflammasome activation and high levels of IL‐1α production in plasmacytoid dendritic cells (pDCs) can facilitate lung cancer cell proliferation.^91^ Similarly, AIM2 in DCs may play an important role in host immune response and tumor microenvironment.

### 3.3 AIM2 in neutrophils

Neutrophils are swiftly attracted to areas of cell or tissue injury upon infection or inflammation and release multiple cytokines, including IL‐1β.^92^, ^93^, ^94^ Key components of the AIM2 inflammasome complexes are highly expressed, mainly in the cytoplasm of neutrophils.^38^ However, the precise relationship between the AIM2 inflammasome and proinflammatory cytokine secretion in neutrophils is poorly understood.

### 3.4 AIM2 in adaptive immune cells

T cell reactivity against AIM2 has been found in patients with melanoma, suggesting that AIM2‐derived peptides are an ideal candidate for immunomonitoring.^95^ Indeed, as a sensor of DNA, AIM2 induces an antigen‐specific antibody response and is used as an adjuvant to enhance therapeutic efficacy through CD8^+^ T cell adaptive immunity.^39^, ^40^, ^41^, ^42^ For example, AIM2 promotes the multifunctional CD8^+^ T cell activation elicited by the viral capsid protein 1 vaccine,^39^ which favors long‐lasting protection against *Coxsackievirus* B_3_‐induced myocarditis.^40^ Similarly, AIM2‐adjuvant vaccines exhibit antitumor therapeutic efficacy by heightening tumor‐specific CD8^+^ T cell immunity.^41^ The involvement of AIM2 in vaccine‐induced immune responses requires type I IFN signaling via cGAS‐independent STING‐IRF7 signaling.^96^ Interestingly, antigen‐specific adaptive immune responses are dramatically decreased in Aim2^−/−^ mice after DNA vaccination, which is independent of IL‐1β and IL‐18.^42^

A recently published paper in Nature shows that AIM2 is expressed at a much higher level in Treg cells than in innate immune cells, in both mice and humans, and can be induced by TGFβ. A high level of AIM2 can maintain the normal function of Treg cells and protect mice from developing autoimmune encephalomyelitis and inflammatory colitis by reducing AKT–mTOR signaling.^43^ These findings indicating the inflammasome‐independent role of AIM2 in adaptive cells differ from AIM2's classic function in innate immunity.

Additionally, Aim2^−/−^ mice have an increased frequency of gastric CD8^+^ T_RM_ cells, whereas an elevated production of CXCL16 in B cells contributes to the suppression of homing receptors.^44^ This result indicated that highly expressed AIM2 in gastric B cells may inhibit CXCL16 production in the control of infiltration and retention of CD8^+^ T cells within chronic inflammatory tissues, which is independent of inflammasome and IFN‐β signaling.^44^ Furthermore, preferential expression of AIM2 is found in mature memory CD27^+^ B cells of adults.^45^ However, AIM2 is directly suppressed by transcription factor FOXP1 in mature human B cells.^97^ To date, the role of AIM2 in B cells is largely unknown.

### 3.5 AIM2 in autoimmune diseases

One of the hallmarks of SLE is dysregulation of type I IFN signaling.^98^ Recent studies have revealed that mouse p202 and human IFI16‐β impede AIM2 inflammasome formation and stimulate IFN‐β production.^84^, ^99^, ^100^, ^101^ An altered AIM2 inflammasome system together with other IFN‐inducible protein‐mediated responses may trigger the pathogenesis of SLE. In line with this hypothesis, treatment of murine macrophages with IFN‐α differentially modulates the levels of AIM2 and p202.^99^ Notably, reduced levels of AIM2 within immune cells as well as high production of p202 and IFN‐β have been described in lupus‐prone strains of mice.^99^, ^102^ Further studies have indicated that activation of the IRF5‐Blimp‐1‐p202 pathway increases SLE susceptibility,^103^ possibly by affecting B cell differentiation.^104^ B cell‐activating factor (BAFF), which is highly expressed in circulating CD3^+^ T cells and SLE patients' serum, decreased the expression of AIM2 but increased the levels of p202.^105^, ^106^ Therefore, the imbalance between AIM2 regulation and the type I IFN pathway leads to the progress of SLE.

Constitutive levels of AIM2 are higher in the livers, kidneys, and PBMCs of MRL/LPR mice than wild mice.^107^ A reduction in AIM2 expression is found in macrophages isolated from female SLE patients versus sex‐matched healthy individuals.^108^ A reduction in DNA methylation of AIM2 was identified in SLE patients in comparison with their healthy siblings.^109^ All these findings suggest that organ‐, cell type‐, and sex‐dependent expression and epigenetic changes in AIM2 are related to the occurrence of SLE.^99^, ^109^, ^110^ Besides, defective clearance of apoptotic cell debris and aberrant activation of macrophages have been proposed to facilitate the progression of SLE.^98^ Indeed, increased activation of AIM2 inflammasome has been found in unstimulated macrophages collected from male SLE patients,^108^ leading to abnormal macrophage maturation and thereby contributing to the immune dysregulation of severe lupus nephritis.^7^ Abnormal AIM2 activation in macrophages thereby contributes to disease severity of SLE.

Additionally, abundant cytosolic DNA can induce AIM2‐dependent release of IL‐18 and IL‐1β in keratinocytes and ductal salivary epithelia that contributes to the pathogenesis of psoriasis and primary Sjogren's syndrome, respectively.^9^, ^111^ Intriguingly, the antibacterial peptide LL‐37 interacts with DNA to prevent AIM2 inflammasome overactivation and IL‐1β oversecretion in psoriatic skin.^111^, ^112^ The silencing of AIM2 leads to inflammasome inactivation, inhibition of macrophage infiltration, and reduced signs of polyarthritis in self‐DNA‐driven chronic polyarthritis model DNase II^−/−^ mice.^11^, ^33^, ^113^ Overall, dysregulation of the AIM2 response to self‐derived dsDNA plays a role as a key driver of autoimmune diseases.

## 4 ROLE OF AIM2 IN CANCERS

### 4.1 AIM2 in melanomas

The introduction of normal human chromosome 6 can inhibit human malignant melanoma growth rate, and restrain the tumorigenicity of nude mice.^114^ Further studies showed that the expression of AIM2 was related to the inhibition of melanoma phenotype.^1^ AIM2 is constitutively expressed in melanocytes from normal skin.^115^ An increase in AIM2 expression occurs in common melanocytic nevi and most primary melanomas, whereas AIM2 expression is generally low or even nonexistent in melanoma metastases.^116^ The upregulation of AIM2 can reverse malignant properties (Table 2).^1^ AIM2 was initially demonstrated as a tumor‐suppressive factor in the control of tumorigenicity in melanoma.

**Table 2:** Role of AIM2 in cancers

Cancers	AIM2 expression	Role of AIM2	Mechanism of AIM2 role	References
Melanoma	↓	Tumor‐suppressive	–	1, 116
		Proliferation↓ Metastasis↓		
NSCLC	↑	Tumor‐promoting	Regulation of inflammasome pathways (the IL‐1β/STAT3 pathway)	12, 91, 117, 118, 119
		Proliferation↑		
		EMT↑	Regulation of mitochondrial dynamics	
		Metastasis↑	Induction of high levels of IL‐1α secretion from pDCs	
Colon cancer	↓	Tumor‐suppressive	Regulation in an inflammasome‐independent manner (suppression of DNA‐PK‐mediated AKT activation and suppression of the PI3K/AKT pathway)	13, 120, 121, 122, 123, 124, 125, 126
		Proliferation↓ Apoptosis↑		
		EMT↓		
		Intestinal stem cell proliferation		
		Effects on the gut microbiota	Regulation of inflammasome pathways	
Hepatocellular carcinoma	↓	Tumor‐suppressive	Regulation of inflammasome pathways (suppression of the mTOR‐S6K1 pathway and regulation of the HBx/AIM2/FN1 signaling axis)	127, 128
		Proliferation↓ Metastasis↓		
		EMT↓ Liver inflammation		
		Proliferative responses		
Renal carcinoma	↓	Tumor‐suppressive	Regulation of inflammasome pathways (induction of autophagy‐related gene expression)	15, 129
		Proliferation↓ Apoptosis↑		
		Autophagy↑ Metastasis↓		
Breast cancer	↓	Tumor‐suppressive	Antagonization of NF‐κB transcriptional activity (suppression of antiapoptotic protein expression)	16, 77, 130, 131, 132
		Proliferation↓ Apoptosis↑		
		Metastasis↓	Regulation of TRAIL‐expressing hMSCs through secretion of IFN‐β	
		EMT↓	Immunosuppression of macrophages	
Prostate cancer	↓	Tumor‐suppressive	Induction of loss of IFN signaling	133, 134
HPV‐infected cervical carcinoma	↓	Tumor‐suppression	Regulation of inflammasome pathways	17, 135
		Pyroptosis↑	(SIRT1–AIM2 axis)	
SSC	↑	Tumor‐promoting	Regulation of inflammasome pathways	19, 20
		Proliferation↑ Apoptosis↓		
		EMT↑	Activation of NF‐κB signaling (induction of cell cycle regulatory genes expression)	
		Metastasis↑		
EBV‐associated NPC	↑	Tumor‐promoting	Regulation of inflammasome pathways	^21^
		Proliferation↑		
		Neutrophil recruitment		

Abbreviations: ↑, significantly higher; ↓, significantly lower; AIM2, absent in melanoma 2; EBV, Epstein–Barr virus; EMT, epithelial–mesenchymal transition; hMSCs, human mesenchymal stem/stromal cells; HPV, human papillomavirus; IFN, interferon; NF‐κB, nuclear factor κB; NPC, nasopharyngeal carcinoma; NSCLC, non‐small‐cell lung cancer; SCC, squamous cell carcinoma; TRAIL, TNF‐related apoptosis‐inducing ligand.

### 4.2 AIM2 in NSCLC

**Figure 3:**
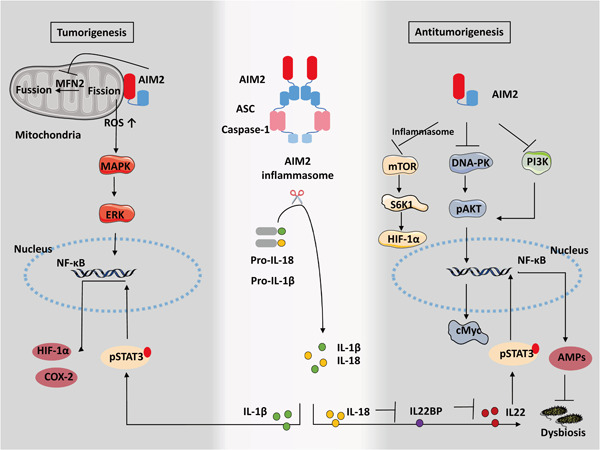
Tumorigenesis and antitumorigenesis of AIM2 in mammary tumors. The mature IL‐1β driven by AIM2 inflammasome promotes HIF‐1α expression through the NF‐κB/COX‐2 pathway. The association between mitochondria and AIM2 contributes to the MAPK/ERK signaling response that leads to tumorigenesis in NSCLC. In contrast, in colon cancer, DNA‐PK, as well as PI3K, are inhibited by AIM2, triggering the inactivation of AKT and c‐Myc, thereby preventing tumorigenesis. Additionally, the mTOR‐S6K1‐HIF‐1α pathway is inhibited by the AIM2 inflammasome, which plays an antitumorigenic role in hepatocellular carcinoma. AIM2, absent in melanoma 2; ASC, apoptosis‐associated speck‐like protein containing a CARD; dsDNA, double stranded DNA; GSDMD, gasdermin‐D; IFN, interferon; IL, interleukin

Dysregulation of inflammatory cytokines triggered by activation of inflammasomes in the lung is reported to promote lung tumorigenesis.^136^, ^137^ Considerable evidence has identified the carcinogenic role of highly expressed AIM2 in NSCLC (Figure 3).^12^, ^138^ Strong expression of AIM2 significantly increases cell viability, migration, and invasion in an inflammasome‐dependent way, whereas the silencing of AIM2 can suppress cell proliferation and result in G2/M phase accumulation.^12^ IL‐1β upregulates hypoxia‐induced factor‐1α (HIF‐1α) that promotes tumor growth and metastasis through the NF‐kB‐dependent cyclooxygenases‐2 (COX‐2) pathway, whereas depleting IL‐1β reverses the malignant phenotype via dephosphorylation of STAT3.^117^, ^138^, ^139^ Also, AIM2 colocalizes with mitochondria, promoting tumor growth by regulating mitochondrial dynamics.^118^ Increased mitochondrial fission induced by AIM2 causes downregulation of fusion‐related protein MFN2 and increases cellular reactive oxygen species^140^ responses, thereby contributing to the MAPK/ERK pathway activation.^118^ Thus, AIM2 in NSCLC exerts tumor‐promoting effects in an inflammasome‐dependent manner and regulation of mitochondrial dynamics, presumably triggering an IL‐1β/STAT3 response by the nuclear factor‐κB (NF‐κB)/COX‐2/HIF‐1α pathway and contributing to the MAPK/ERK pathway activation.

A higher percentage of cancer‐derived pDCs, which do not manifest cytotoxic activity but instead facilitate cancer cell growth,^119^ are detected in NSCLC tumors than in control tissues.^91^ Surprisingly, the AIM2 inflammasome is highly expressed in pDCs, which enhances calcium efflux and reactive oxygen species (ROS) release from mitochondria, resulting in calpain activation and IL‐1α release, thereby promoting tumor proliferation.^91^ Therefore, inhibiting the expression of AIM2 in NSCLC may pave a new way for the treatment of NSCLC.

Epithelial–mesenchymal transition (EMT) is considered one of the essential steps in the progression of malignant tumors.^141^ Activation of the AIM2 inflammasome promotes EMT in lung cancer.^119^ The antitumor drug luteolin downregulates the expression of AIM2 and inhibits EMT of NSCLC.^142^

### 4.3 AIM2 in colon cancer

Increased expression of AIM2 is detected in inflammatory bowel diseases,^143^ but many of the AIM2 alterations that have been reported in patients with colon cancer involve a lack or even complete loss of AIM2 expression.^144^, ^145^ Several previous studies have analyzed the link between AIM2 expression and colon cancer progression. Surprisingly, the absence of AIM2 is closely related to poor clinicopathological features and prognosis.^120^, ^121^, ^146^ Similarly, many tumors are found in AIM2‐deficient mice.^13^ This suggests that AIM2 is required to restrain the progression of colon cancer.

It has been revealed the AIM2 inflammasome in colonic epithelial cells is activated and contributes to the maintenance of intestinal integrity against dysbiosis. The AIM2 inflammasome executes its function by regulating the IL‐18/IL‐22BP/IL‐22 pathway and the levels of specific antimicrobial peptides (AMPs).^122^ Genomic deletion of AIM2 in colon cancer contributes to DNA‐dependent protein kinase (DNA‐PK)‐induced AKT overactivation, thereby enhancing cell survival,^13^ whereas AIM2 restoration promotes cell cycle G2/M arrest and prevents tumor cell proliferation and viability through suppression of DNA‐PK‐mediated AKT activation independent of the inflammasome.^13^, ^123^, ^124^, ^125^ Also, AIM2 contributes to tumor apoptosis by inhibiting the PI3K/AKT pathway.^126^ Noticeably, AIM2 upregulation blocks EMT‐mediated cell migration and invasion in a manner that is dependent on AKT and inflammasome pathways.^124^, ^147^ Interestingly, AIM2 also restricts intestinal stem cell proliferation and expansion by dampening phosphorylation of AKT and cMyc activation, as evidenced by the great number of proliferating tumor‐initiating stem cells seen without AIM2.^125^ Surprisingly, the decrease in AIM2 in intestinal epithelial cells is accompanied by overexpression of AIM2 in infiltrating immune cells that possibly leads to deleterious protumorigenic responses or angiogenesis.^121^ Additionally, AIM2^−/−^ mice that are hyper susceptible to tumorigenesis of colon cancer are aggravated by dysbiotic gut microbiota, but an exchange of the dysbiotic gut microbiota with that of healthy mice ameliorates this increased susceptibility.^122^, ^148^ Therefore, AIM2 plays a protective role in colon cancer. It exerts function by inflammasome‐independent manner, control of intestinal stem cell proliferation, and regulation of gut microbiota.

### 4.4 AIM2 in hepatocellular carcinoma

AIM2 expression is noticeably reduced in hepatocellular carcinoma patients and significantly associated with poor overall survival.^14^, ^127^ Activation of the AIM2 inflammasome triggers pyroptosis and suppresses cell proliferation and invasion, thereby inhibiting tumorigenicity in nude mice through antagonism of the mTOR‐S6K1‐HIF‐1α signaling axis.^14^ Additionally, hepatitis B virus X protein (HBx)‐mediated loss of AIM2 is correlated with a high tendency for metastasis and activation of the EMT process in HBV‐related hepatocellular carcinoma tissues, which is mediated by fibronectin 1 (FN1) expression.^127^ Consequently, DHA, as an autophagy promoter that triggers ROS‐induced nuclear and mitochondrial DNA damage, suppresses cell proliferation through the AIM2/caspase‐1 inflammasome complex.^149^ In contrast, another study showed that AIM2 responds by promoting inflammation and proliferative responses during tumor initiation in an inflammasome‐dependent manner.^128^ Elevated AIM2 in Kupffer cells promotes inflammation during carcinogenic liver injury.^128^ Accordingly, the genetic deletion of AIM2 protected against tissue damage and cancer progression in the diethylnitrosamine‐induced hepatocellular carcinoma model.^128^ This implies two contrasting roles of AIM2, which may be caused by different models or different disease stages.

### 4.5 AIM2 in renal carcinoma

Necrotic cell DNA can induce AIM2 inflammasome activation in macrophages that contributes to the progression of chronic kidney disease.^150^ In contrast, AIM2 expression is dramatically decreased in renal carcinoma patients.^15^, ^129^ Restoration of AIM2 inhibits tumor proliferation, migration, and invasion while enhancing cell apoptosis by inducing autophagy‐related gene expression.^15^ Additionally, the DNA vaccine‐containing renal carcinoma specific antigen carbonic anhydrase IX and AIM2 adjuvant, which promotes cells overexpressing inflammasome components and proinflammatory cytokines, can prevent tumor growth.^41^, ^129^ Thus, AIM2 is regarded as a tumor‐impressive factor and used as an adjuvant to attenuate renal carcinoma.

### 4.6 AIM2 in breast cancer

Activation of AIM2 drives apoptosis and suppresses proliferation through antagonizing NF‐κB transcriptional activity and inhibiting antiapoptotic protein expression, thereby restraining mammary tumor growth in vivo.^16^ Besides, elevated levels of AIM2 in the tumor stroma can also suppress tumor cells in TRAIL‐sensitive triple‐negative breast cancers.^130^, ^151^ TRAIL‐expressing human mesenchymal stem/stromal cells (hMSCs) promote apoptosis and inhibit metastasis of breast cancer cells (MDA cells) after TNF‐α treatment by secreting IFN‐β. This occurs in an AIM2‐dependent manner triggered by DNA fragments from apoptotic cells.^152^, ^153^ Apoptotic cell‐derived DNA fragments sensed by AIM2 further increase TRAIL levels in hMSCs, resulting in feed‐forward stimulation and increased apoptosis of MDA cells.^153^ Considering that TRAIL is modulated by type I IFNs and induces apoptosis during microbial infections,^154^ both AIM2 and type I IFNs likely participate in the tumor‐suppressive microenvironment. Additionally, cancer‐associated fibroblasts (CAFs) exhibit functional similarities to hMSCs, indicating that hMSCs transition into CAFs.^151^, ^153^ A further understanding of how AIM2 mediates TRAIL upregulation upon exposure to cytosolic DNA fragments will provide a novel therapeutic approach for TRAIL‐sensitive cancer.

Cancerous cell‐free DNA (cfDNA) molecules promote tumor progression and resistance to anticancer therapies.^131^ Ribosomal cfDNA (cf‐rDNA) exists in circulating cfDNA in breast cancer patients.^155^ Exposure to extracellular cf‐rDNA molecules stimulates the survival of tumor cells, represses AIM2 expression, and reduces apoptosis, thereby facilitating tumor malignancy by triggering TLR9‐MyD88‐NF‐kB signaling.^132^ Taken together, these findings show that AIM2 exerts tumor suppression in breast cancer.

### 4.7 AIM2 in prostate cancer

Increased AIM2 expression and IL‐1β production in senescent prostate epithelial cells induce benign prostatic hyperplasia,^156^, ^157^ whereas decreased AIM2 expression is accompanied by the tumorigenesis of prostate cancer, which is characterized by the loss of IFN signaling.^133^, ^134^ IFN stimulation robustly induces the AIM2 inflammasome in prostate tumors.^18^ Additionally, NLRP12 is significantly increased, which may promote tumor growth by triggering NF‐κB and IL‐1β signaling.^157^ Further work is needed to investigate the potential function of these sensors in the control of prostate diseases.

### 4.8 AIM2 in HPV‐infected cervical cancer

The AIM2 inflammasome is known to trigger pyroptosis in response to DNA viruses^4^, ^135^; for example, AIM2 is activated against HPV16 infection in keratinocytes.^135^ However, AIM2 expression is inhibited by the deacetylase Sirtuin 1 (SIRT1) through the destabilization of RELB messenger RNA in HPV‐infected cervical cancer, assisting HPV‐infected tumors in escaping antiviral immunity.^17^ Pyroptosis triggered in SIRT1‐knockdown cells can be transmitted to naïve tumor cells via the intercellular transmission of the AIM2 inflammasome,^4^, ^135^ whereas pyroptotic death signaling can be prevented by SIRT1 restoration.^17^ This implies AIM2 inflammasome plays the tumor‐suppressive role in HPV‐infected cervical cancer.

### 4.9 AIM2 in SCC

The high levels of AIM2 expression in intestinal epithelial cells in response to pathogenic infections indirectly inhibit AKT activation, reducing stem cell proliferation in colonic tumors.^125^ In the epidermis, the AIM2 inflammasome promotes wound repair in the skin recovering from inflammation, which is accompanied by a high level of pAKT and proliferating epithelial stem cells.^158^ However, this imbalance may lead to uncontrolled proliferative disease. Indeed, the AIM2 gene is overexpressed in both cutaneous and oral SCC.^20^, ^159^ Increased activation of the AIM2 inflammasome promotes cell viability and restrains apoptosis by inducing cell cycle regulatory gene expression in cutaneous SCC^20^ or by activating NF‐κB signaling in p53‐deficient oral SCC.^159^ Additionally, high levels of AIM2 are associated with strong tumor invasion through upregulation of the production of the invasion‐related proteinases MMP1 and MMP13 in cutaneous SCC.^20^ Likewise, a high metastatic capacity of oral SCC cells is associated with increased EMT.^19^ Depletion of AIM2 inhibits tumor growth and vascularization of SCC in vivo^20^ and therefore AIM2 is a potential oncogenic driver in SCC.

### 4.10 AIM2 in EBV‐associated NPC

In EBV‐associated NPC, EBV and irradiation‐induced AIM2 inflammasome activation lead to mature IL‐1β release that promotes tumor proliferation.^21^ Interestingly, when the IL‐1β level reaches a certain threshold, this effect can be reversed by the recruitment of immunostimulatory tumor‐associated neutrophils.^21^, ^160^ This implies that immune cells can be recruited by tumor cells to control the host response through activation of the AIM2 inflammasome.^21^

## 5 AIM2 SERVES AS A THERAPEUTIC TARGET?

In the past decade, several groups have attempted to identify and develop treatment strategies by targeting AIM2. A series of animal experiments have been reported, especially in cancers (Table 3).

**Table 3:** Animal data targeting AIM2

Disease	Approach	References
Polyarthritis	Silence of AIM2 reduced signs of polyarthritis in self‐DNA driven chronic polyarthritis model DNase II^−/−^mice	^11^
NSCLC	Downregulation of AIM2 mediated by luteolin reduced tumorigenicity in the A549 and H460 xenograft mouse models	^142^
Colon cancer	Reciprocal exchange of gut microbiota with wild‐type mice reduced colorectal tumorigenesis in Aim2‐deficient mice	125, 126, 147
Hepatocellular carcinoma	Knockdown of AIM2 reduced tissue damage and cancer progression in the diethylnitrosamine‐induced hepatocellular carcinoma mode	^128^
Renal carcinoma	H1/pAIM2 nanoparticles attenuated tumor growth in 786‐O‐xenograft mice	41, 161

Abbreviations: AIM2, absent in melanoma 2; NSCLC, non‐small‐cell lung cancer.

The traditional drug luteolin, which was developed as a treatment for NSCLC, was found to decrease the AIM2 inflammasome to therapeutic effect.^149^ Recently, promoting an adaptive response with an AIM2‐adjuvanted vaccine has demonstrated therapeutic efficacy, relieving the symptoms of coxsackievirus B_3_‐induced myocarditis and renal carcinoma.^40^, ^129^ Thus, AIM2 is a potential therapeutic target for cancer treatment. However, the utility of this therapeutic strategy will vary depending on the function of AIM2 in different tumors. For example, gene‐based restoration of AIM2 in colon cancer may be a novel approach to cure AIM2‐deficient cancers by restraining DNA‐PK and PI3K,^13^ whereas downregulation of AIM2 leads to inhibition of AIM2‐activated tumors. Furthermore, the tumor‐promoting effects of the AIM2 inflammasome in EBV‐associated NPC can be reversed by neutrophil recruitment upon irradiation.^21^ However, irradiation‐induced nuclear gene damage can increase the potential risks of pneumonitis and chronic fibrosis via AIM2 inflammasome‐mediated cell death.^32^ Similarly, chemotherapy‐induced intestinal toxicity is caused by AIM2 inflammasome activation.^162^ Additionally, macrophages can be transformed into an immunosuppressive phenotype in a process mediated by the AIM2 inflammasome during antibody treatment.^77^, ^163^ The role of the AIM2 inflammasome in autoimmune disease and cancers is complex and multifaceted and more research is needed to clarify these relationships and interactions.

## 6 CONCLUSIONS AND PERSPECTIVES

In recent years, the underlying mechanism by which AIM2 inflammasome affects the innate immune response has been extensively studied. However, the effect of AIM2 in T and B cells remains unclear, which be a shortage if AIM2 is used as a therapeutic target but the current study cannot provide an overall knowledge of AIM2 in the immune system. Therefore, future study is needed to address this issue. Also, AIM2 displays either tumor‐suppressive function or tumorigenesis effects in different cancers. The contrasting roles of AIM2 in cancer are dependent on the model, the disease, and even the stage of the disease. However, AIM2 exerts an important role in the regulation of tumor local microenvironment. Further studies are needed to elucidate the exact mechanism by which self‐derived DNA triggers AIM2 activation and the interactions of AIM2 with other sensor pathways.

## AUTHOR CONTRIBUTIONS

Huan Zhu wrote the manuscript, Christopher Chang, Vera Chan, and Ming Zhao did the editing, Haijing Wu and Qianjin Lu revised the manuscript.

## CONFLICT OF INTERESTS

The authors declare that there are no conflicts of interests.
